# Polydatin alleviates mitochondrial damage and apoptosis of lung epithelial cells by inhibiting toll‐like receptor 4‐dependent macrophage activation in asthma

**DOI:** 10.1002/ame2.70100

**Published:** 2025-12-18

**Authors:** Guangxing Li, Ruobai Liu, Chang Xu, Jianing Yang, Yilan Song, Li Li, Jingzhi Jiang, Liangchang Li, Chongyang Wang, Guanghai Yan

**Affiliations:** ^1^ Jilin Key Laboratory for Immune and Targeting Research on Common Allergic Diseases Yanbian University Yanji P.R. China; ^2^ Department of Anatomy, Histology and Embryology Yanbian University Medical College Yanji P.R. China; ^3^ Key Laboratory of Natural Medicines of the Changbai Mountain, Ministry of Education Yanbian University Yanji P.R. China

**Keywords:** cell–cell cross‐talk, NOD‐like receptor protein (NLRP3) inflammasome, ovalbumin (OVA) stimulation, toll‐like receptor 4 (TLR4)/P2X7R synergy

## Abstract

**Background:**

This study investigated the role of polydatin in regulating macrophage–epithelial cell (EC) interactions during asthma. An asthma model was induced in BALB/c mice using ovalbumin (20 μg).

**Methods:**

The therapeutic effects of polydatin (20 and 40 mg/kg) were evaluated in this asthmatic mouse model. To assess the underlying mechanisms, Bronchial Epithelium Adenovirus 12‐SV40 2B (BEAS‐2B) cells were cocultured with Tohoku Hospital for Pediatrics‐1 (THP‐1) macrophages, in which toll‐like receptor 4 (TLR4) was either overexpressed or knocked down, and subsequently stimulated with lipopolysaccharide (LPS) and ATP. THP‐1 cells underwent a 1‐h pretreatment with polydatin (50 and 100 μmol/L), Class Lipid Inhibitor‐095 (CLI‐095, TLR4 inhibitor, 1 μg/mL), or A438079 (P2X7R antagonist, 10 μmol/L) prior to LPS/ATP challenge.

**Results:**

Findings from Western blotting, enzyme‐linked immunosorbent assay, flow cytometry, real‐time polymerase chain reaction, and immunofluorescence assays demonstrated that modulating TLR4 expression significantly altered interleukin‐1β (IL‐1β) secretion from THP‐1 macrophages and mitochondrial reactive oxygen species (mtROS) production in BEAS‐2B ECs. In the mouse asthma model, polydatin significantly alleviated airway inflammation, oxidative stress, and apoptosis, likely by interfering with TLR4/P2X7R‐mediated signaling and suppressing the activation of the NOD‐like receptor protein inflammasome. Additionally, polydatin significantly reduced IL‐1β and IL‐18 levels and inhibited the infiltration of macrophages and eosinophils. Correspondingly, polydatin significantly attenuated TLR4/P2X7R signaling in THP‐1 cells stimulated with ATP and LPS, thereby reducing IL‐1β and IL‐18 secretion, calcium influx, mtROS production, and apoptosis in BEAS‐2B ECs.

**Conclusions:**

Polydatin is a promising therapeutic candidate for asthma, possibly by targeting macrophage–epithelium cross‐talk via the TLR4/P2X7R axis. Future formulations as capsules or sprays may effectively alleviate airway inflammation and remodeling.

## Graphical abstract

The alleviation of asthma by polydatin is dependent on the blockage of the toll‐like receptor 4 (TLR4)/P2X7R synergy in macrophages. The blockage of the TLR4/P2X7R synergy results in decreased release and secretion of interleukin‐1β (IL‐1β) and IL‐18. In epithelial cells, low IL‐1β and IL‐18 levels inhibit mitochondrial damage and apoptosis.

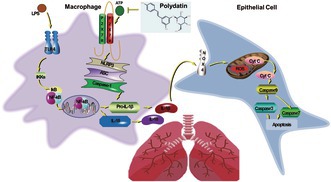

## 1 BACKGROUND

Asthma is a heterogeneous disease with a primary clinical manifestation of chronic airway inflammation. At present, over 300 million people worldwide are diagnosed with asthma, with a yearly increasing incidence and mortality.^1^ Clinically, patients with mild‐to‐moderate asthma can be treated with glucocorticoids, but patients with severe asthma often have symptoms of chest tightness and dyspnea and must be hospitalized. Due to the refractory and relapsing nature of asthma, patients need long‐term standardized management, frequent hospitalization, and continuous medication, which increases the psychological and economic burden. Therefore, it is crucial to explore specific strategies to prevent and treat asthma.

Disturbance of mitochondrial energy metabolism caused by allergens and air pollutants is one of the etiologies of asthma.^2^ The mitochondrion is the major site where endogenous reactive oxygen species (ROS) are produced and is the primary target of ROS. Mitochondrial dysfunction in airways directly leads to the escape of mitochondrial reactive oxygen species (mtROS) from the oxidative respiratory chain, thereby enhancing the sensitivity of airway epithelial cells (AEC) to oxidants and increasing asthma susceptibility.^3^ Therefore, mtROS‐mediated mitochondrial damage plays an important role in asthma pathogenesis.

Approximately 70% of pulmonary immune cells are macrophages, which significantly contribute to airway inflammation characteristic of asthma. Particularly, inflammatory mediators secreted by M2 macrophages induce epithelial cell (EC) injury and apoptosis.^4^ AECs form the primary defensive barrier of the respiratory tract and actively respond to endogenous oxidative damage. Disruption of this epithelial barrier typically coincides with epithelial homeostasis disruption, recruitment and activation of inflammatory cells, as well as polarization toward T‐helper type 2 immunity, ultimately culminating in airway hyperresponsiveness (AHR) and structural remodeling.^5^ Therefore, interactions between macrophages and AECs are crucial in asthma pathogenesis.

Toll‐like receptor 4 (TLR4) is abundantly expressed on pulmonary ECs, playing a critical role in innate immune responses and substantially influencing asthma progression.^6^ Functionally, TLR4 recognizes ligands such as lipopolysaccharide (LPS) and heat shock proteins, and ligand engagement activates the nuclear factor kappa B (NF‐κB) pathway in an MyD88‐dependent manner, thereby enhancing the production and secretion of pro‐inflammatory cytokines.^7^ In addition, TLR4‐mediated activation results in increased secretion of chemokines and adhesion molecules, thereby recruiting macrophages to inflammatory sites. Macrophages primarily secrete cytokines. Simultaneous overexpression of TLR ligands and activation of the purinergic receptor P2X7 (P2X7R) function in concert as a secondary signaling mechanism promoting maturation and release of interleukin‐1β (IL‐1β) and IL‐18.^8^ Besides, ATP is the main energy source that maintains cellular metabolic function under physiological conditions and can be released into the extracellular space as a damage signal molecule for the regulation of various cellular effects under stress conditions.^9^ In asthma, ATP activates the macrophage purinergic receptor P2X7 to promote inflammatory responses in AECs.^10^ Also, the interaction between the P2X7R and the NLRP3 (NOD‐like receptor protein) inflammasome enables ATP to open potassium channels after P2X7R activation, and the efflux of potassium ions activates NLRP3, which ultimately promotes the inflammatory response.^8^ Therefore, the TLR4 and P2X7R signaling greatly affects asthma pathogenesis.

Polydatin is an antioxidant and a natural precursor of resveratrol. It is one of the natural active components from the dried rhizomes of *Reynoutria japonica* Houtt., a traditional Chinese medicine, and also exists in grapes, red wine, hops, peanuts, cocoa products, and mulberry.^11^ Polydatin has many pharmacological effects, such as antioxidation,^12^ anti‐inflammation,^11^ antiallergy,^13^ and ischemia–reperfusion injury resistant activity.^14^ Additionally, polydatin could promote and increase the activation and expression of sirtuin‐1, thereby preventing mitogen‐activated protein kinase p38 from being phosphorylated, cleaved caspase‐3/9 from being activated, and endoplasmic reticulum stress–related proteins from being expressed, resulting in reduced ROS in the mitochondria of rat neurons and weakened mitochondrial damage.^15^ Furthermore, in renal cardiomyocytes and cancer cells, polydatin inhibited myocardial injury and chemoresistance by downregulating NF‐κB, NLRP3 inflammasome, cytokines, and chemokines.^12^ Polydatin could protect against asthma by promoting Nrf2‐mediated antioxidation.^13^ Moreover, polydatin pretreatment significantly inhibited mitochondrial morphology changes, maintained mitochondrial function, and suppressed apoptosis.^14^ However, the antiasthma mechanism of polydatin involving TLR4/P2X7R cross‐talk and mitochondrial damage remains undetermined.

Here, the effects and mechanisms of polydatin in asthma were investigated in vivo using ovalbumin (OVA)–induced asthma mice and in vitro using THP‐1 and BEAS‐2B cells. Changes in inflammatory cells, airway resistance, ROS production, and apoptosis protein in the lungs as well as TLR4/NF‐κB/P2X7R/NLRP3 pathway proteins were determined. In addition, macrophages and airway epithelium cross‐talk were demonstrated after silencing and overexpressing TLR4 in macrophages. Finally, we explored how polydatin mediated the cross‐talk between TLR4 and P2X7R. Our findings possibly provide a new target for diagnosing and treating asthma in clinical practice.

## 2 METHODS

### 2.1 Animals

Forty female BALB/c mice (6–8 weeks old) were obtained from the Animal Research Center at Yanbian University and housed in a pathogen‐free environment. The Yanbian University Ethics Committee approved the study (clearance number: YB.No20210630b040, and approval date: July 15, 2021). All experimental procedures were conducted in accordance with the approved protocol, and the reports were in compliance with the ARRIVE standards.

### 2.2 Animal grouping and treatment

BALB/c mice (*n* = 40) were allocated into five experimental groups, each comprising eight animals. The murine asthma model was developed according to previously established protocols.^16^ Particularly, mice allocated to OVA groups were administered subcutaneous injections containing 20 μg of OVA antigen (Sigma‐Aldrich, CAS: 276889‐40‐8, product A5503) emulsified with 2 mg of aluminum hydroxide adjuvant (InvivoGen, CAS: 21645‐51‐2). This antigen‐adjuvant formulation was adjusted to 200 μL and injected on days 0, 7, and 14. From 14 to 21 days post‐OVA, mice in the PD‐20‐OVA group were administered 20 mg/kg of polydatin (CAS: 65914‐17‐2, 15721, Sigma‐Aldrich) intraperitoneally daily for eight consecutive days, whereas those in the PD‐40‐OVA group received 40 mg/kg of the drug via the same route and schedule. Dexamethasone (CAS: 50‐02‐2, D1756, 1 mg/kg, Sigma), as a positive control, was administered using the same protocol as polydatin. The control group was administered normal saline. From days 21 to 23, the OVA treatment groups underwent aerosolized 5% OVA treatment for three consecutive days.

### 2.3 AHR detection

The animals were anesthetized with pentobarbital sodium (100 mg/kg, CAS: 57‐33‐0, 2018042001, Huaxia Chemical Reagent, Chengdu, China) on day 24. The mouse tracheas were cannulated and ventilated at 1.47 mmHg positive end‐expiratory pressure, and subsequently, the methacholine challenge was performed. After treatment with 5, 10, and 30 mg/mL of aerosol methacholine, lung resistance was determined with lung function invasive measurement using the FlexiVent Pulmonary System (SCIREQ, Beijing GYD Labtech Co., Ltd., China). The three highest dynamic resistance values were averaged to represent AHR.

### 2.4 Sample collection

Twenty‐four hours after the final challenge, the mice were intraperitoneally anesthetized with sodium pentobarbital (50 mg/kg). Approximately 5 min later, the mice lost consciousness and were subsequently killed via cervical dislocation. The lungs were washed twice with 0.7 mL of phosphate‐buffered saline to collect the bronchoalveolar lavage fluids (BALF). Then, the left and right lungs underwent pathological analysis and protein and RNA extraction, respectively.

### 2.5 Histopathological analysis of lung

Samples were stained with hematoxylin–eosin (HE) and periodic acid–Schiff for detecting pulmonary inflammation and goblet cell hyperplasia, as previously described.^16^

### 2.6 Immunofluorescence and immunohistochemistry staining

Lung tissues collected for immunostaining procedures were first fixed and permeabilized. After overnight incubation (~20 h) at 4℃, the specimens were treated with primary antibodies targeting TLR4, P2X7R, and F4/80 proteins. These antibodies were obtained from Affinity Biosciences, Thermo Fisher Scientific, and Abcam, respectively. Subsequently, tissue sections were incubated for 2 h with secondary antibodies. Additionally, immunohistochemical analyses involved primary antibodies specific for cleaved caspase‐3 (Cell Signaling Technology, 9661) and cleaved caspase‐9 (Affinity Biosciences, AF5240), followed by suitable secondary antibodies. Sections were counterstained with hematoxylin and subsequently analyzed microscopically.

### 2.7 Terminal deoxynucleotidyl transferase dUTP nick‐end labeling staining

Apoptosis in lung tissue sections was assessed utilizing a terminal deoxynucleotidyl transferase dUTP nick‐end labeling (TUNEL) assay (Beyotime Biotech, C1089). Paraffin‐embedded tissue sections were initially processed by dewaxing and rehydration, and then incubated with DNase‐free proteinase K at 37℃ for half an hour. Then, the sections were treated with 50 μL of the prepared TUNEL reaction mixture and were further incubated for 1 h at ambient temperature. Fluorescence intensity was quantified via the Cytation 5 imaging system (BioTek Instruments, USA).

### 2.8 Flow cytometry

Eosinophil populations within BALF were quantified using flow cytometry after labeling cells with CD45.2 antibody with allophycocyanin (BD Pharmingen, 558702) and Siglec‐F antibody with phycoerythrin (BD Pharmingen, 552126). Postfixation, cell suspensions underwent flow cytometric analyses utilizing a Cytoflex cytometer (Beckman Coulter, Miami, FL, USA).

### 2.9 Real‐time quantitative polymerase chain reaction

Total RNA was isolated using the RNA Easy Kit (Invitrogen) and then subjected to accurate RNA quantification using a METASH UV spectrophotometer (Shanghai, China), ensuring sample purity at an approximate A260/A280 absorbance ratio of 2.0. For complementary DNA synthesis, each 25‐μL reaction contained 1 μL of RNA, Oligo(dT) 15 primers, and AMV Reverse Transcriptase enzyme obtained from Takara. Real‐time quantitative polymerase chain reaction (RT‐qPCR) was conducted on the Azure cielo 6 PCR platform employing a three‐step SYBR Green RT‐PCR kit (KR123, Tiangen). Amplification efficiencies consistently approached 100%, with standard curve correlation coefficients (*R* ^2^) typically ~0.99. Glyceraldehyde‐3‐phosphate dehydrogenase (GAPDH) served as the endogenous reference gene.

### 2.10 Enzyme‐linked immunosorbent assay

Concentrations of IL‐1β and IL‐18 in BALF and culture supernatants were quantified using commercial enzyme‐linked immunosorbent assay (ELISA) kits (MLB00C and DY7625‐05, R&D Systems, Minneapolis) following previously established methodologies.^16^ Additional cytokines were measured in BALF using ELISA kits provided by Enzyme Linked Biotechnology (Shanghai, China, catalog numbers: ml063583, ml063156, ml063157, and mIC50278‐1).

### 2.11 Cell culture and treatment

Human THP‐1 cells (Shanghai Cytobiology Research Institute) were cultured in RPMI‐1640 medium (C3001‐0500, Vivacell), supplemented with 10% fetal bovine serum (FBS) and 1% penicillin–streptomycin. THP‐1 cells were differentiated into macrophages via Phorbol 12‐myristate 13‐acetate (PMA) exposure before subsequent experimentation. Additionally, BEAS‐2B cells were maintained in Dulbecco's Modified Eagle Medium (DMEM, C3110‐0500, Vivacell) with 10% FBS and 1% PS. THP‐1‐derived macrophages were stimulated with 1 μg/mL of LPS for 4 h and then exposed to ATP (3 mmol/L) for 30 min. Prior to LPS and ATP stimulation, cells were pretreated for 1 h with polydatin (50 or 100 μmol/L), CLI‐095 (a TLR4‐specific inhibitor, CAS: 243984‐11‐4, 1 μg/mL, InvivoGen), or A438079 (a P2X7R‐specific antagonist, CAS: 899507‐36‐9, 10 μmol/L, Abcam).

### 2.12 Cell transfection

THP‐1 macrophages underwent transfection procedures using Lipofectamine 2000 reagent (11 668 500, Invitrogen). Cells were treated with plasmids encoding scrambled control small interfering RNA (siRNA), TLR4‐specific siRNA, negative control plasmid (cytomegalovirus [CMV]), or CMV‐TLR4 plasmid (for TLR4 overexpression, GeneChem, Shanghai, China) for 72 h. After transfection, cells were stimulated with 1 μg/mL of LPS for 4 h and then treated with ATP (3 mmol/L) stimulation for 30 min. The human‐specific TLR4 siRNA sequences employed were as follows: forward, 5′‐GGAAUGAGCUAGUAAAGAATT‐3′; reverse, 5′‐UUCUUUACUAGCUCAUUCCTT‐3′.

### 2.13 Coculture of THP‐1 and BEAS‐2B cells

Interactions between PMA‐induced THP‐1 macrophages and BEAS‐2B cells, along with the role of TLR4 signaling in mtROS production and apoptosis induction within BEAS‐2B cells, were examined using a Transwell coculture system. Particularly, the system utilized six‐well Transwell inserts equipped with a 0.4‐μm porous polycarbonate membrane (Corning Inc., USA), which allows the diffusion of soluble factors while preventing direct cell–cell contact between the two cell types. BEAS‐2B ECs were seeded into the upper compartment of the six‐well Transwell apparatus containing DMEM, whereas THP‐1 cells, incubated in RPMI‐1640 medium, were activated with LPS and ATP in the lower chamber. After a 24‐h incubation period at 37℃, supernatants from cell cultures were harvested for subsequent analysis.

### 2.14 Western blotting

Western blotting (WB) proceeded following the previous description.^16^ The primary antibodies included TLR4 (AF7017, Affinity), p‐IκBα (Ser32, 2859, Cell Signaling Technology, lnc), IκBα (4812, CST), NADPH oxidase 2 (NOX2) (ab129068, Abcam), NADPH oxidase 4 (NOX4) (ab154244, Abcam), NF‐κB p‐P65 (Ser536, 3033, CST), NF‐κB P65 (3034, CST), cytochrome C (Cyt C, ab133504, Abcam), cleaved caspase‐3 (Asp175, 9661, CST), cleaved caspase‐9 (Asp353, AF5240, Affinity), P2X7R (77665, Thermo), NLRP3 (MA523919, Thermo), Apoptosis‐Associated Speck‐like Protein Containing a CARD (ASC, 67824, CST), caspase‐1 (ab1872, Abcam), IL‐1β (12703, CST), IL‐18 (57058, CTS), cleaved‐IL‐1β (Asp116, 83186, CST), and GAPDH (5174, CST). Image J software was used for protein blot analysis.

### 2.15 Assessment of ROS and mtROS in cells and lung tissues

Intracellular ROS and mtROS levels were assessed using mitochondrial‐specific oxidative‐sensitive fluorescent probes: H2DCFDA (S0033S, Beyotime Biotech) and MitoSOX Red (M36008, Thermo Fisher Scientific). Cells were treated with 5 μmol/L 2',7'‐Dichlorodihydrofluorescein diacetate (DCFH‐DA) or 5 μmol/L MitoSOX dye for 10 min at 37℃. Dihydroethidium (DHE, 10 μmol/L, C3807, Apexbio, Houston, TX, USA) was incubated with lung tissue samples for 10 min at 37℃ to detect ROS in nature. A Cytation 5 imaging apparatus (BioTek) was used for quantitative measurement of fluorescence intensity.

### 2.16 Analysis of calcium concentration

The calcium concentration in THP‐1 cells was measured using Fluo 3‐AM (F8840, Solarbio, Beijing, China). Cells were cultured for 1 h in Fluo 3‐AM working solution (5 μL) at 37℃. After the cells were cleaned using HEPES (4‐(2‐hydroxyethyl)‐1‐piperazineethanesulfonic acid) buffer, 1% HBSS solution of FBS was added, and the mixture was cultured half an hour at 37℃. The fluorescence intensity measurement was obtained using Cytation 5.

### 2.17 Detection of mitochondrial membrane potential

Cells were treated with JC‐1 (5 μmol/L, C2006, Beyotime) for 15 min at 37℃ and then observed using Cytation 5. We computed the mitochondrial depolarization degree based on the red–green fluorescence ratio and then normalized it to the control group.

### 2.18 Statistical analysis

Data were statistically processed using GraphPad Prism (version 7.0). Results were reported as mean values accompanied by standard errors of the mean obtained from three separate experimental repeats. Differences between experimental groups were statistically assessed by performing analysis of variance (ANOVA) and then Dunnett's post hoc test for multiple comparisons. For nonparametric data that did not meet the normality assumption, we applied the Wilcoxon rank‐sum test for pairwise comparisons. *p* < 0.05 was considered statistically significant.

## 3 RESULTS

### 3.1 TLR4 of macrophages regulates mitochondrial damage and inflammation in ECs through cell‐to‐cell cross‐talk

**FIGURE 1:**
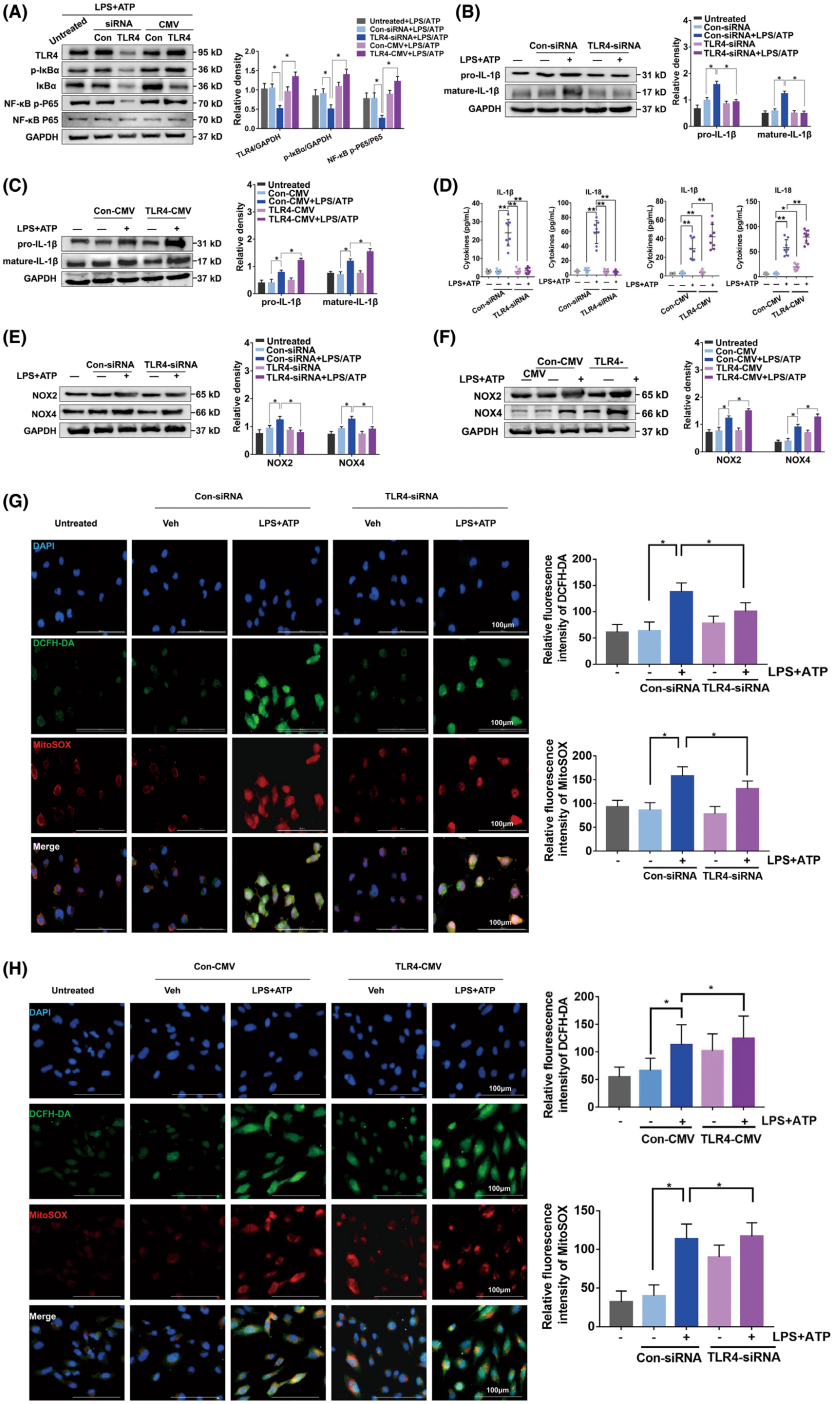
TLR4 (toll‐like receptor 4) is indispensable in promoting IL‐1β (interleukin‐1β) secretion from LPS (lipopolysaccharide) + ATP‐activated macrophages. THP‐1 cells were transfected with siRNA (small interfering RNA) or CMV (cytomegalovirus) plasmid to achieve TLR4 knockdown or overexpression, and treated with LPS (1 μg/mL) for 4 h and ATP (3 mmol/L) for 30 min successively. (A) WB (Western blotting) measured TLR4, IκBα, NF‐κB (nuclear factor kappa B) P65, and phosphorylation of IκBα and NF‐κB P65 in macrophages. WB measured pro‐IL‐1β and mature‐IL‐1β levels in macrophages after (B) TLR4 knockdown and after (C) TLR4 overexpression. (D) ELISA (enzyme‐linked immunosorbent assay) measured the protein expression of IL‐1β (interleukin‐1β) and IL‐18 in supernatant (*n* = 8). WB was used for measuring NOX2 (NADPH oxidase 2) and NOX4 (NADPH oxidase 4) in ECs (epithelial cells) after (E) TLR4 knockdown and after (F) TLR4 overexpression. Representative fluorescence of mtROS (mitochondrial reactive oxygen species) with MitoSOX in ECs after (G) TLR4 knockdown and after (H) TLR4 overexpression. Data in the histograms were obtained from three independent experiments, and data presentation followed the mean ± SEM (standard error of the mean) format. ANOVA (analysis of variance) was used for the multiple comparisons, and Dunnett's post hoc test (A–C and E–H) or Wilcoxon rank‐sum test (D) was performed subsequently. *p < 0.05 and **p < 0.01.

We knocked down TLR4 with siRNA or overexpressed TLR4 in THP‐1 cells stimulated with PMA. According to WB analysis, TLR4 knockdown made IκBα and NF‐κB P65 less phosphorylated in LPS‐ and ATP‐treated cells (Figure 1A). In contrast, TLR4 overexpression significantly promoted IκBα and NF‐κB P65 phosphorylation (Figure 1A). Furthermore, WB analysis found that LPS + ATP stimulation upregulated pro‐IL‐1β and mature‐IL‐1β expressions, whereas the expressions were significantly suppressed by the knockdown of TLR4 (Figure 1B). In contrast, TLR4 overexpression promoted IL‐1β (Figure 1C). Overall, TLR4 is needed for the release of IL‐1β in THP‐1 cells using LPS/ATP as stimuli.

IL‐1β mediated the NOX4/ROS/TXNIP/NLRP3 pathway through a positive feedback loop in a diabetic retinopathy cell model.^17^ Numerous studies have shown that cells secrete not only IL‐1β but also IL‐18 through activating LPS‐mediated TLR4/MYD88/NF‐κB/NLRP3 pathway, and IL‐18 also crucially regulates NOX2 and NOX4.^18^, ^19^ According to ELISA results, knockdown and overexpression of TLR4 significantly affected the levels of the two inflammatory cytokines in the culture supernatant (Figure 1D). Furthermore, WB revealed the upregulation of NOX2 and NOX4 due to LPS + ATP stimulation, whereas knockdown of TLR4 suppressed the expression of NOX2 and NOX4 (Figure 1E). In contrast, overexpression of TLR4 enhanced the expression of NOX2 and NOX4 (Figure 1F).

Furthermore, to confirm the impact of TLR4 on mtROS and total ROS in the BEAS‐2B EC line, we cocultured ECs with macrophages that had TLR4 knockdown or overexpression. After treatment with LPS + ATP, we detected mtROS and total ROS using MitoSOX and DCFH‐DA in ECs, finding the obvious reduction in mtROS and ROS in ECs after coculture with the LPS + ATP–treated macrophage with TLR4 knockdown (Figure 1G). However, TLR4 overexpression resulted in a significant upregulation of mtROS and total ROS (Figure 1H). These results implied that macrophage‐derived IL‐18 and IL‐1β potentially impacted NOX/ROS signaling in ECs and that TLR4 may play a key role in this process.

### 3.2 Polydatin decreases ROS and inflammation in asthmatic mice

**FIGURE 2:**
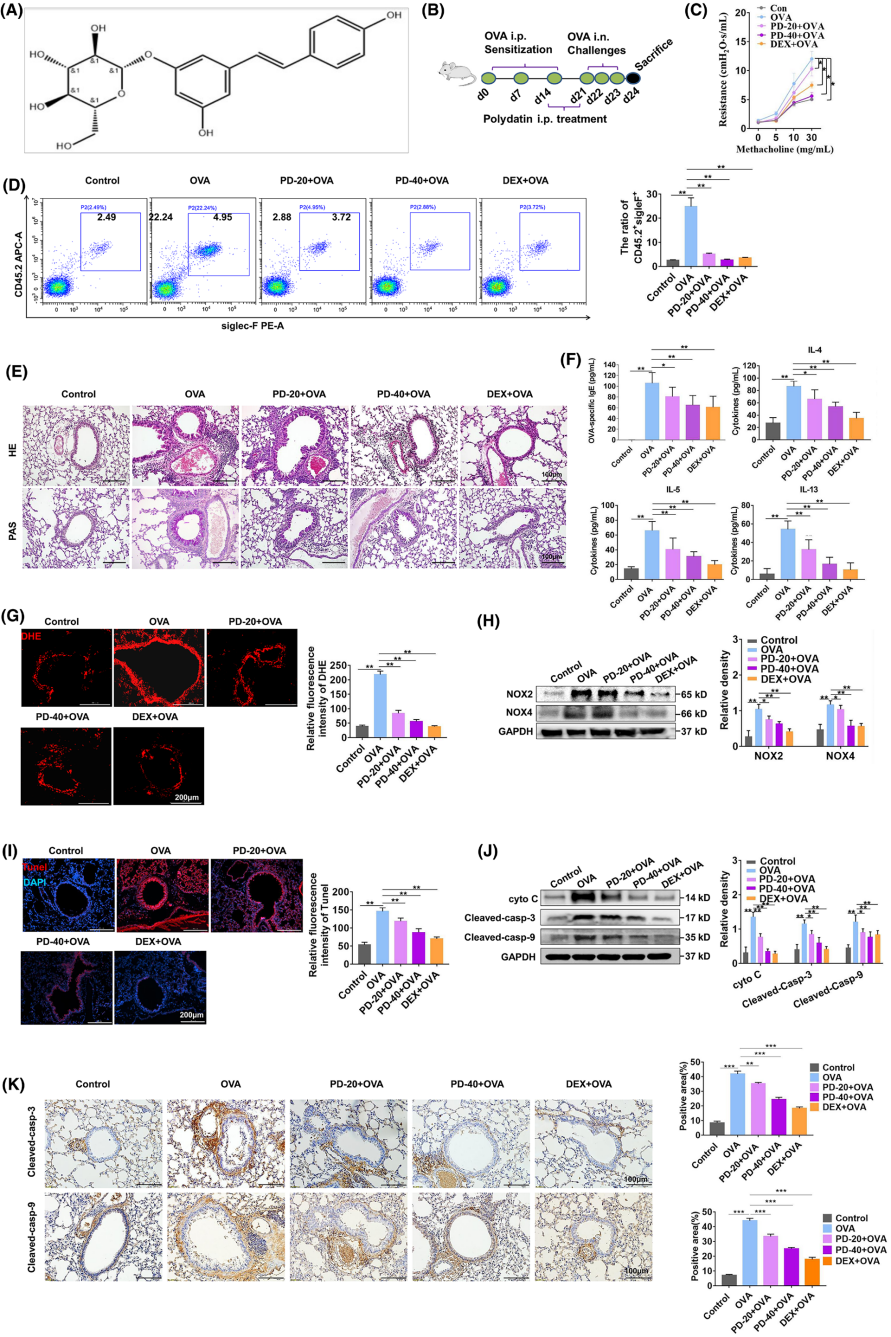
Polydatin relieves ROS (reactive oxygen species) and inflammation in asthmatic mice. To investigate asthma pathology, we developed an asthma mouse model via sensitization of BALB/c mice with ovalbumin (OVA). After initial OVA exposure, animals received intraperitoneal injections of polydatin prior to subsequent aerosolized OVA challenges. (A) Structural representation of polydatin. (B) Experimental protocol diagram illustrating timeline and treatment schedules. (C) Lung resistance alterations in response to methacholine stimulation (*n* = 8 per group). (D) Quantification of eosinophils (identified as CD45.2^+^ Siglec‐F^+^ cells) within bronchoalveolar lavage fluid (BALF) using flow cytometry. (E) Representative histological lung sections stained with hematoxylin–eosin (HE) and periodic acid–Schiff (PAS). (F) Measurement of cytokine concentrations in BALF using ELISA (enzyme‐linked immunosorbent assay) assays (*n* = 8 per group). (G) Fluorescent microscopic images demonstrating ROS (reactive oxygen species) production via dihydroethidium (DHE) staining in lung tissues. (H) WB (Western blotting) quantification of NOX2 (NADPH oxidase 2) and NOX4 (NADPH oxidase 4) protein expression in pulmonary tissues. (I) Identification of apoptotic cells using TUNEL (terminal deoxynucleotidyl transferase dUTP nick‐end labeling) staining in lung sections with corresponding quantitative analysis of positive cells (*n* = 3). (J) WB detection of apoptosis‐related proteins within lung homogenates. (K) Immunohistochemical staining depicting the expression and localization of cleaved caspase‐3 and cleaved caspase‐9 proteins (*n* = 3). *p < 0.05, **p < 0.01, and ***p < 0.001.

The study evaluated how polydatin affected OVA‐induced AHR, ROS, and lung inflammation by establishing the mouse model of asthma. Figure 2A shows the polydatin chemical structure and Figure 2B the establishment procedure of the asthma model. Relative to the control group, OVA treatment increased airway resistance, which was significantly relieved by polydatin (Figure 2C). Next, we adopted the flow cytometry for the detection of the proportion of eosinophils in BALF, finding the obvious reduction in the proportion after polydatin treatment (Figure 2D). Furthermore, polydatin inhibited OVA‐induced peribronchial inflammation, accumulation of eosinophils, and proliferation of goblet cells (Figure 2E). Additionally, we found that treatment with polydatin efficiently prevented IL‐4, IL‐5, IL‐13, and IgE from being produced in BALF induced by OVA (Figure 2F). We further explored whether polydatin could inhibit oxidative stress in asthmatic mice by detecting ROS by DHE staining. Relative to the control group, OVA increased ROS production in the lung tissue, whereas ROS production was significantly weakened by polydatin treatment (Figure 2G). Next, we conducted WB to examine NOX2 and NOX4 expressions, finding that polydatin treatment reversed the upregulated NOX2 and NOX4 induced by OVA (Figure 2H). The accumulation of ROS induced by IL‐1β and IL‐18 triggers oxidative stress and is involved in apoptosis.^20^, ^21^ We performed a TUNEL assay as well as WB and immunohistochemical analysis of apoptosis proteins, investigating the effects of polydatin on apoptosis in asthmatic lung tissue. We found that polydatin decreased apoptosis (Figure 2I) and apoptosis proteins, including Cyt C, cleaved caspase‐3, and cleaved caspase‐9 in asthmatic lung tissue (Figure 2J,K).

### 3.3 Polydatin suppresses TLR4/P2X7R/NLRP3 signal axis in OVA‐induced asthma

**FIGURE 3:**
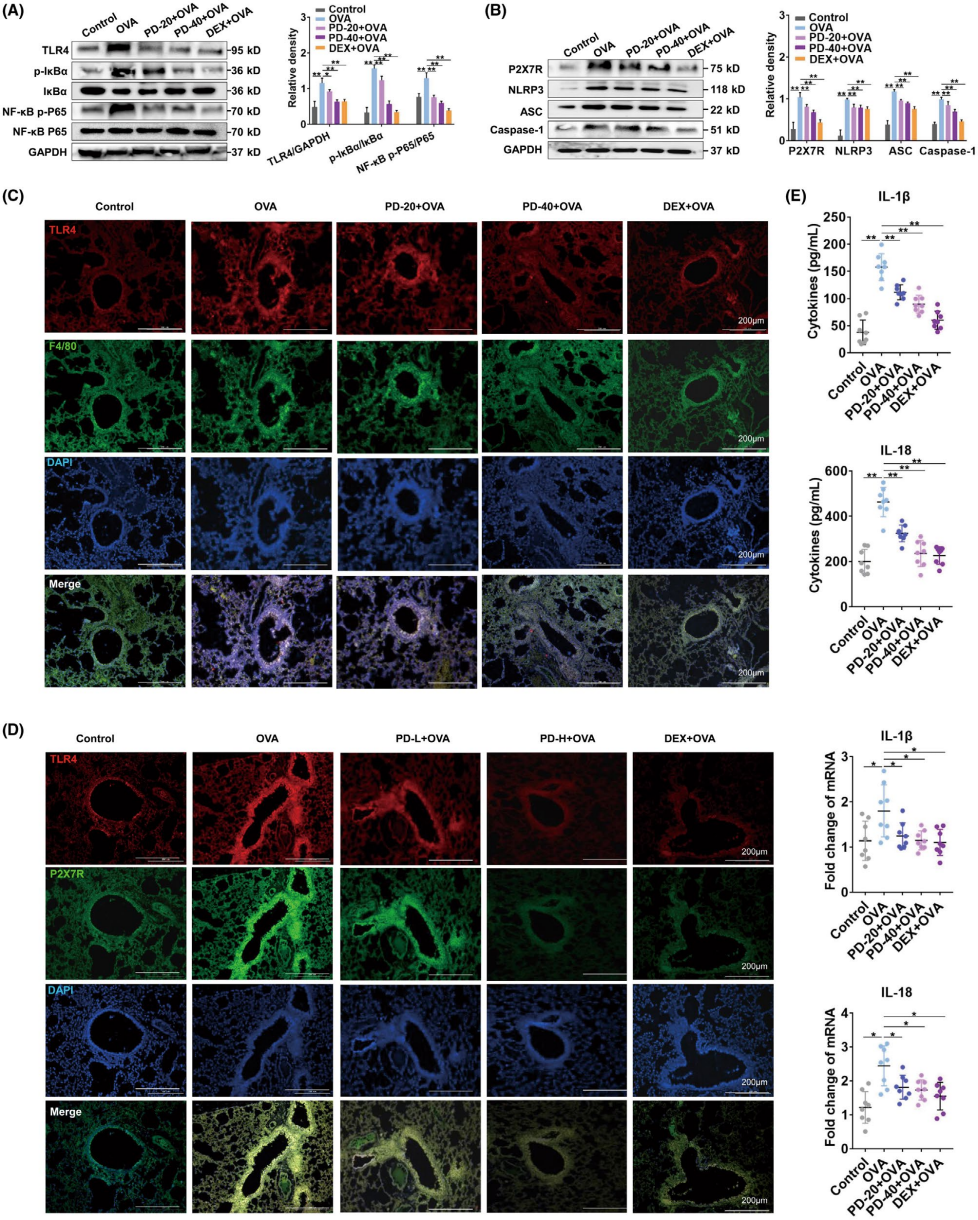
Polydatin suppresses TLR4 (toll‐like receptor 4)/P2X7R/NLRP3 (NOD‐like receptor protein) activation in OVA (ovalbumin)–induced asthma. WB (Western blotting) measured (A) TLR4, IκBα, NF‐κB (nuclear factor kappa B) P65, and phosphorylation of IκBα and NF‐κB P65, and (B) P2X7R, NLRP3, ASC, and caspase‐1 in lung tissues. (C) Co‐expression of F4/80 (green) and (D) P2X7R (green) with TLR4 (red) in mouse lung tissues was assessed via immunofluorescence staining combined (*n* = 3). (E) ELISA (enzyme‐linked immunosorbent assay) and real‐time PCR (polymerase chain reaction) were used to measure IL‐1β (interleukin‐1β) and IL‐18 protein levels in BALF (bronchoalveolar lavage fluid) and mRNA (messenger RNA) expression (*n* = 8), and Dunnett's post hoc test (A–D) or Wilcoxon rank‐sum test (E) was performed subsequently. *p < 0.05 and **p < 0.01.

WB revealed that polydatin treatment significantly suppressed the levels of pivotal proteins in TLR4/NF‐κB and P2X7R/NLRP3 signaling axes compared with the OVA model group (Figure 3A,B). Immunofluorescence assays (Figure 3C,D) further demonstrated that polydatin suppressed the activation of both TLR4 and P2X7R in lung ECs and macrophages within the pulmonary tissues. To further explore the mechanisms underlying polydatin's anti‐inflammatory effects in asthmatic mice, real‐time PCR and ELISA analyses targeting IL‐1β and IL‐18 were conducted. Polydatin administration significantly reduced both IL‐1β and IL‐18 messenger RNA expression in pulmonary tissues, as well as their corresponding protein secretion into BALF (Figure 3E). Collectively, these results suggest that polydatin may mitigate mitochondrial impairment and apoptosis in asthma through inhibition of the TLR4/P2X7R/NLRP3 pathway.

### 3.4 Polydatin inhibits IL‐1β and IL‐18 via blocking TLR4/P2X7R in macrophages

**FIGURE 4:**
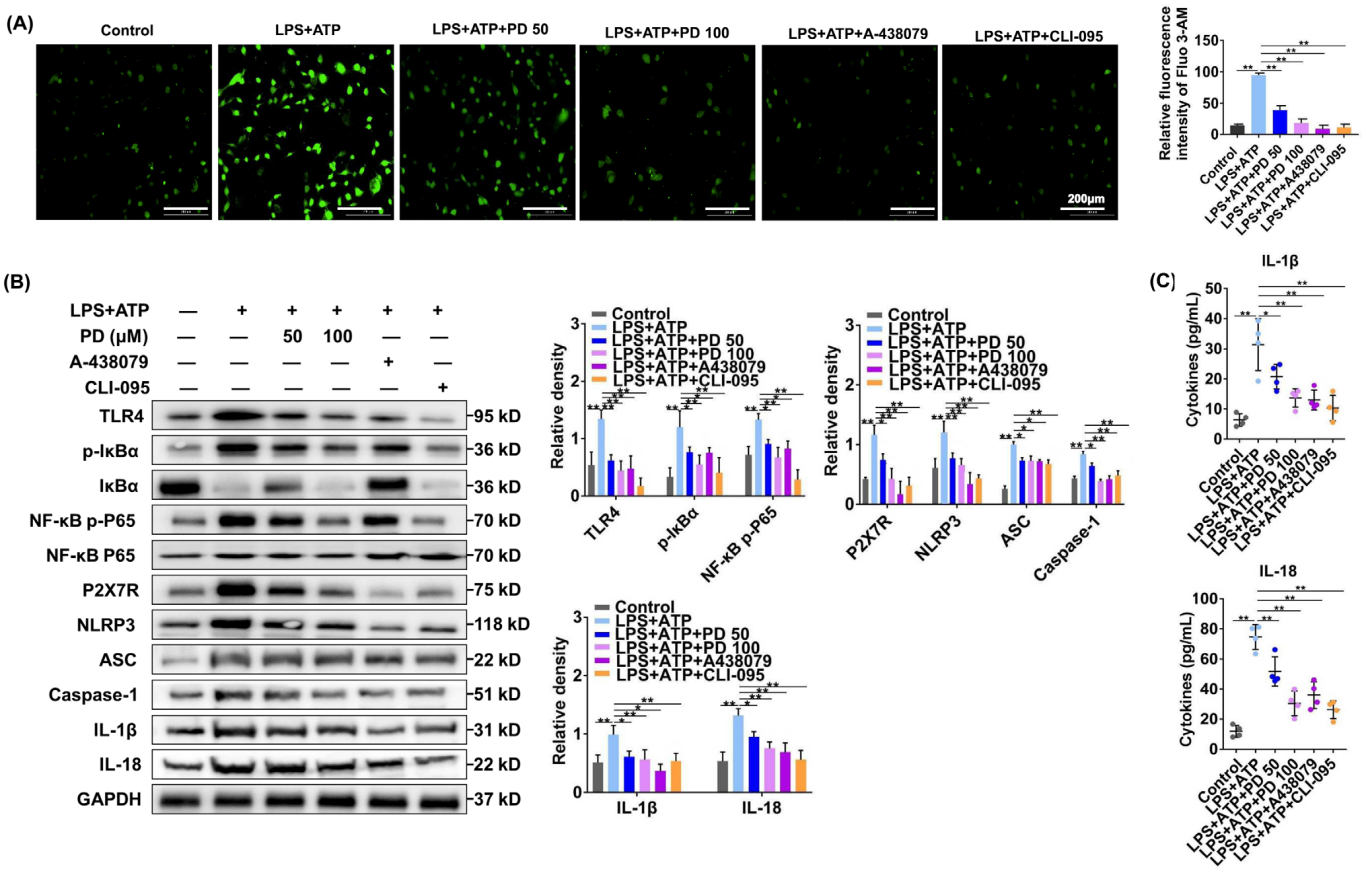
Polydatin inhibits IL‐1β (interleukin‐1β) and IL‐18 by blocking the TLR4 (toll‐like receptor 4)/P2X7R pathway in macrophages. (A) Intracellular calcium concentrations were analyzed utilizing Fluo 3‐AM fluorescent staining (*n* = 3). (B) WB (Western blotting) was performed to quantify the levels of TLR4, IκBα, NF‐κB (nuclear factor kappa B) p65, phosphorylated forms of IκBα and NF‐κB p65, P2X7R, NLRP3 (NOD‐like receptor protein) inflammasome components, IL‐1β, and IL‐18 (*n* = 3). (C) Macrophage‐derived protein secretion levels of IL‐1β and IL‐18 were evaluated using ELISA (enzyme‐linked immunosorbent assay). (A and B) or Wilcoxon rank‐sum testing (C). *p < 0.05 and **p < 0.01.

We further treated cells with CLI‐095 (a TLR4 inhibitor) or A438079 (a P2X7R antagonist) in the presence of LPS and ATP, confirming the role of TLR4/P2X7R cross‐talk. Then, the calcium concentrations within macrophages were analyzed. LPS + ATP treatment resulted in obviously increased intracellular calcium concentration, although the concentration was significantly decreased after treatment with polydatin, A438079, and CLI‐095 (Figure 4A). The results suggest that there is cross‐talk between P2X7R and TLR4. Additionally, WB results indicated a pronounced upregulation of TLR4/NF‐κB pathway proteins, P2X7R, components of the NLRP3 inflammasome, and IL‐1β and IL‐18 after combined LPS and ATP stimulation (Figure 4B). However, treatment with CLI‐095 (a TLR4 inhibitor) or A438079 (a selective antagonist of P2X7R) significantly downregulated the expression of TLR4, P2X7R, IL‐1β, and IL‐18; reduced the levels of NF‐κB p65 and IκBα; and inhibited the activation of the NLRP3 inflammasome. Similarly, polydatin suppressed signaling via TLR4 and P2X7R, and subsequently inhibited NLRP3 inflammasome activity, leading to decreased secretion of IL‐1β and IL‐18 into the culture medium (Figure 4C). Therefore, polydatin effectively diminished inflammatory cytokine release by attenuating NLRP3 inflammasome activation via disrupting TLR4 and P2X7R interaction.

### 3.5 Polydatin suppresses ROS production and apoptosis of ECs by blockage of TLR4/P2X7R pathways

**FIGURE 5:**
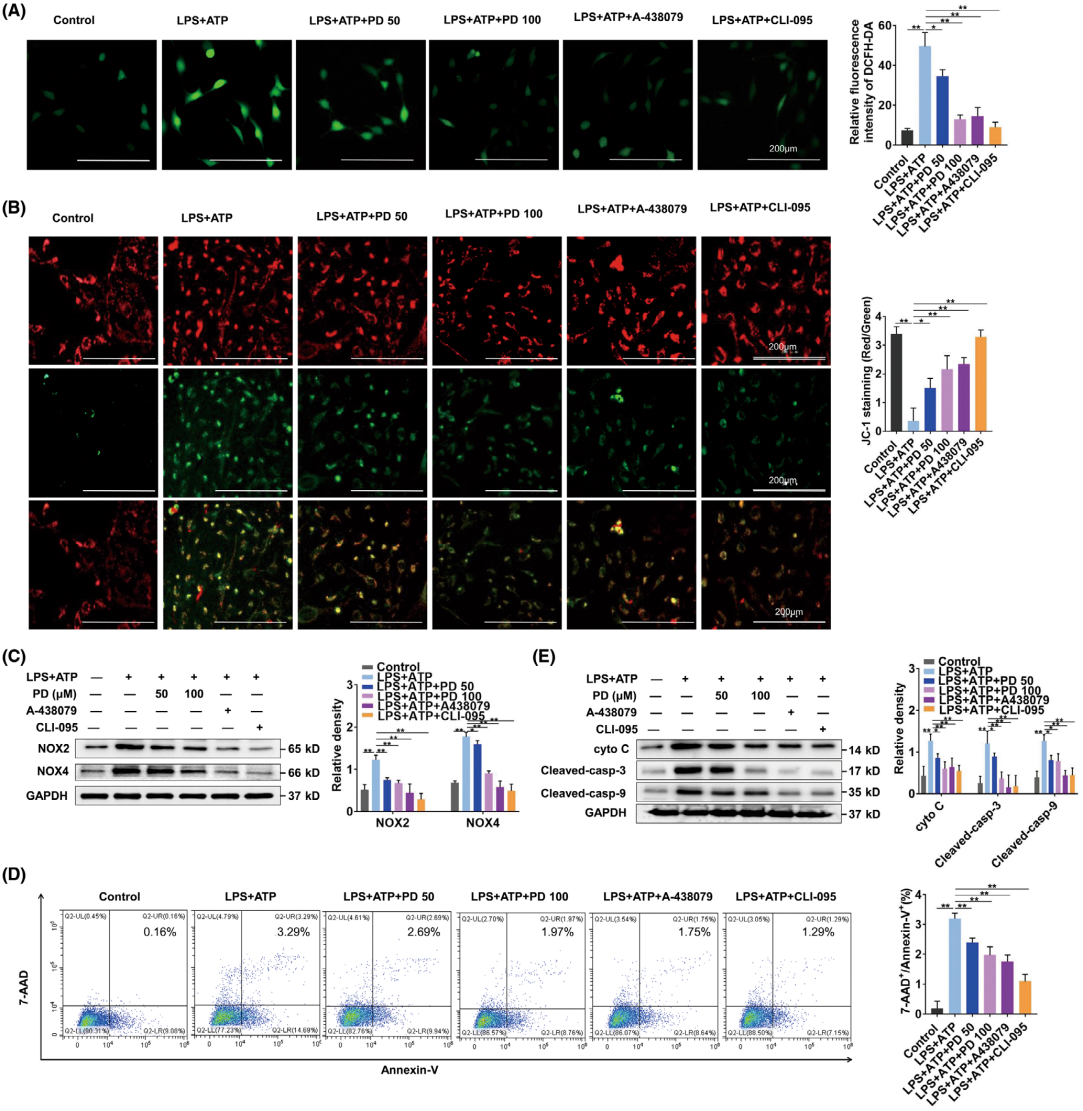
Polydatin suppresses ROS (reactive oxygen species) production and apoptosis of ECs (epithelial cells) by blockage of the TLR4 (toll‐like receptor 4)/P2X7R pathway in macrophages. (A) Intracellular ROS levels in BEAS‐2B cells were determined through DCFH‐DA fluorescence staining (10 μmol/L). (B) Alterations in MMP (mitochondrial membrane potential) were assessed using JC‐1 dye staining. (C) WB (Western blotting) analysis quantified protein expression of NOX2 (NADPH oxidase 2) and NOX4 (NADPH oxidase 4), and proteins related to apoptosis in BEAS‐2B ECs (epithelial cells). (D) Flow cytometry quantified apoptosis based on the proportion of annexin‐V^+^/7‐AAD^+^ positive cells (*n* = 3). (E) Additionally, WB was used to determine apoptosis‐related protein levels in BEAS‐2B cells. Histogram results represent the mean ± SEM (standard error of the mean) from three independent replicates. *p < 0.05 and **p < 0.01.

To verify how polydatin inhibited TLR4/P2X7R cross‐talk, we measured ROS and mitochondrial membrane potential (MMP) in ECs. The results showed that polydatin, CLI‐095, and A438079 treatments all significantly reduced ROS production (Figure 5A) and restored MMP (Figure 5B). WB results showed that polydatin, CLI‐095, and A438079 all significantly reduced the expressions of NOX2 and NOX4 (Figure 5C). We further evaluated the effect of polydatin, CLI‐095, and A438079 on apoptosis of ECs. Flow cytometry analysis showed that treatment with polydatin, CLI‐095, and A438079 significantly inhibited LPS + ATP–induced apoptosis (Figure 5D). According to WB analysis, LPS + ATP led to the upregulation of Cyt C and cleavage of C3 and C9, whereas treatment with polydatin, CLI‐095, and A438079 significantly inhibited their expressions, consistent with the flow cytometry results (Figure 5E). Overall, polydatin may inhibit ROS production and EC apoptosis by inhibiting TLR4 and P2X7R cross‐talk.

## 4 DISCUSSION

Asthma is an autoimmune disease induced by repeated irritation of the airways by allergens. Prolonged inflammation increases airway permeability to bacterial endotoxins, which increases serum LPS levels.^22^ LPS‐stimulated human monocytic THP‐1 cells can be used to investigate the antiasthmatic and anti‐inflammatory mechanisms of conventional drugs in conjunction with OVA‐induced mouse models of asthma.^23^ In addition, sensitized ECs respond to inflammatory cytokines, causing cellular and mitochondrial damage. ATP is released from damaged mitochondria as a second danger signal in asthma progression and promotes IL‐1β release and maturation.^24^ Ultimately, LPS/ATP binding provides two signals that activate NLRP3 inflammasome via TLR4/P2X7R synergy.^8^ Therefore, in this study, we used LPS + ATP as an inducer of macrophages. Our results showed that polydatin inhibited the upregulation of AEC mitochondrial damage and apoptosis by suppression of TLR4 and P2X7R synergy and macrophage‐derived IL‐1β and IL‐18, thus attenuating OVA‐induced asthmatic airway inflammation.

Emerging evidence suggests that therapeutic interventions targeting macrophage–EC interactions may effectively treat or prevent asthma pathogenesis.^25^ Thus, the present study explored the role of TLR4 in macrophage–EC cross‐talk triggered by LPS and ATP stimuli. Macrophages underwent either TLR4 knockdown or overexpression prior to coculturing with ECs exposed to LPS and ATP. Previous reports have demonstrated that TLR4 activation facilitates the maturation of IL‐1β and IL‐18 precursors, contributing substantially to respiratory inflammatory responses.^1^, ^18^, ^26^ Persistent IL‐1β elevation promotes NOX4 expression, triggering subsequent ROS production.^19^ Ultimately, NOX4 aggravates airway inflammation by activating ROS‐mediated EC damage and apoptosis. Our data found that TLR4/NF‐κB activated IL‐1β and IL‐18 secretion by regulating NOX signaling pathways, which indicates the key role of TLR4/NF‐κB in ameliorating mtROS through mediating the cross‐talk of macrophages with ECs.

Polydatin exerts an inhibitory impact on asthma development and progression.^13^ Consistently, our results demonstrate that polydatin suppressed AHR, eosinophilia, and lung inflammation, and attenuated OVA‐induced airway goblet cell hyperplasia in asthmatic mouse lung tissues. It is shown that excessive ROS can cause lung and airway damage and is associated with asthma severity.^27^ Furthermore, persistent ROS can lead to AEC damage and apoptosis, resulting in enhanced secretion of inflammatory cytokines.^28^ Here, polydatin could block OVA‐induced ROS production and apoptosis in lung tissue. Moreover, macrophage infiltration was observed in lung tissues after OVA stimulation and induced TLR4/NF‐κB and P2X7R/NLRP3 signaling pathways to be activated in asthmatic lung tissue. However, polydatin treatment significantly prevented the TLR4/P2X7R pathway from being activated. Additionally, ATP leads to an increase in intracellular Ca^2+^ through activation of P2X7R. Therefore, we measured calcium flux in macrophages. We also found that polydatin inhibited intracellular calcium concentration, as well as the activation of TLR4/P2X7R and its downstream NLRP3 inflammasome. These effects were similar to those of CLI‐095 and A438079. These results suggest that polydatin may be an inhibitor of TLR4/P2X7R cross‐talk.

IL‐1β is an effective pro‐inflammatory cytokine and critical in cell–cell cross‐talk. IL‐1β released by tumor‐associated macrophages could increase the synthesis of Hypoxia‐Inducible Factor 1 Alpha (HIF‐1α) in liver cancer cells and promote cross‐talk between macrophages and tumor cells.^29^ Macrophage‐derived IL‐1β provides a pivotal point for CX3CL1‐CX3CR1 to synergistically promote macrophage–mesothelial cross‐talk.^30^ Moreover, IL‐18 significantly contributes to innate immune activation, driving inflammation and apoptosis at the cellular level.^21^ Therefore, an investigation of the direct effects of IL‐1β and IL‐18 on ECs after their release from macrophages warrants further attention. Our results indicated pronounced secretion of IL‐1β and IL‐18 from activated macrophages, and subsequent coculture with ECs triggered epithelial damage and apoptosis. The underlying molecular mechanisms need to be elucidated. Importantly, we identified that TLR4/P2X7R signaling interactions critically mediated the secretion of these cytokines. Polydatin strongly inhibited the pro‐inflammatory effects mediated by TLR4/P2X7R interactions, significantly reducing IL‐1β and IL‐18 secretion.

Chronic inflammation primarily mediates allergic asthma pathogenesis. Mitochondrial damage can make inflammatory signaling pathways excessively activated, causing chronic inflammation. Mitochondrial damage and dysfunction are closely associated with asthma pathogenesis.^31^ Mitochondria are generally basic organelles containing double separate membranes responsible for the production of endogenous ROS via oxidative phosphorylation. Overloaded ROS accumulation negatively impacts mitochondrial membrane stability, causing MMP loss and Cyt C release, thus activating the mitochondrial apoptosis program.^32^ The present investigation demonstrated mitochondrial dysfunction, alterations in MMP, enhanced mtROS production, and increased apoptosis in ECs after macrophage secretion of IL‐1β and IL‐18. Nevertheless, the precise causal relationship between EC dysfunction and IL‐1β/IL‐18 secretion remains unclear and necessitates further exploration. Additionally, we analyzed NOX2, NOX4, and apoptosis‐associated proteins in BEAS‐2B cells cocultured with macrophages activated by LPS plus ATP. Results indicated that polydatin significantly suppressed NOX enzyme activity, apoptosis rates, caspase‐3/caspase‐9 activation, Cyt C release, and ROS accumulation, consistent with findings obtained from our asthma animal model. Collectively, these data suggest that polydatin ameliorates asthma by restraining macrophage‐mediated activation of the NLRP3/P2X7R signaling axis. Through this inhibition, polydatin attenuates macrophage‐derived IL‐1β and IL‐18 secretion, subsequently reducing mtROS accumulation and apoptosis in AECs.

This study has some limitations. First, although our study provides insights into the therapeutic effects and mechanisms of PD in asthma, specific data on the lung distribution of PD and its pharmacokinetic profiles in various tissues are lacking. Future studies are essential to elucidate the pharmacokinetic parameters of PD, including its tissue distribution, half‐life, and metabolism, which will provide more comprehensive insights into its therapeutic potential in clinical settings. Addressing these pharmacokinetic aspects will be critical for translating our findings into clinical applications and understanding the full impact of PD on asthma and other respiratory conditions.

Additionally, the use of established cell lines (BEAS‐2B and THP‐1‐derived macrophages) represents a key limitation. Although these cell lines offer advantages in phenotypic stability, culture reproducibility, and suitability for mechanistic studies, they cannot fully replicate the physiological complexity of primary cells. For example, BEAS‐2B cells lack the complete differentiation profile of native bronchial ECs, and THP‐1‐derived macrophages may differ from primary alveolar macrophages in cytokine secretion patterns and stimulus responsiveness. Future studies could employ more clinically relevant models, such as humanized mouse models, primary cells from asthmatic patients, or airway organoids, to better validate the translational potential of PD.

Moreover, regarding clinical translation, beyond the aforementioned pharmacokinetic data, the efficacy of PD in different asthma subtypes has not been investigated. Clinical asthma is highly heterogeneous, and PD's therapeutic effects may vary with patient subtypes or comorbidities. Moreover, the synergistic effects of PD with clinically used asthma drugs and potential adverse reactions require further verification.

## 5 CONCLUSIONS

**FIGURE 6:**
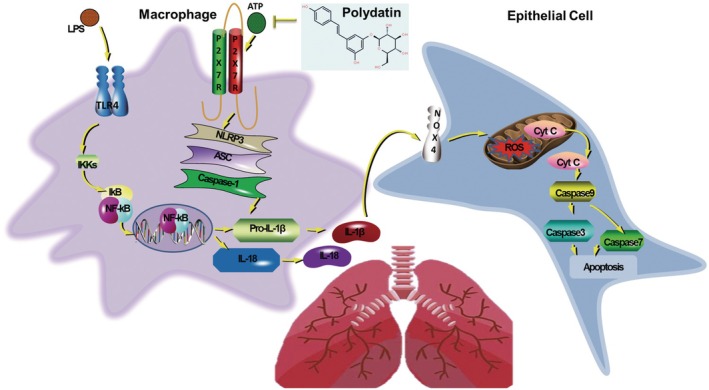
Schematic diagram of the mechanisms underlying the alleviation of OVA (ovalbumin)–induced asthma by polydatin. The alleviation of asthma by polydatin is dependent on the blockage of the TLR4 (toll‐like receptor 4)/P2X7R synergy in macrophages. The blockage of the TLR4/P2X7R synergy results in decreased release and secretion of IL‐1β (interleukin‐1β) and IL‐18In ECs, low IL‐1β and IL‐18 levels inhibit mitochondrial damage and apoptosis.

Overall, polydatin demonstrated a potential therapeutic effect in attenuating airway inflammation, ROS production, and cell apoptosis in asthma models (Figure 6). Treatment with polydatin reduced inflammatory cytokine production and suppressed NLRP3 inflammasome activation in macrophages, as well as inhibited EC mitochondrial damage and apoptosis, thus attenuating asthma. Mechanistically, polydatin exerted its effects by inhibiting the TLR4/P2X7R signaling pathway, cytokine release, and cellular processes related to inflammation and oxidative stress. In the future, polydatin may be a valuable protective agent for asthma treatment.

## AUTHOR CONTRIBUTIONS

**Guangxing Li:** Conceptualization; data curation; funding acquisition; investigation; validation; writing – original draft. **Ruobai Liu:** Conceptualization; data curation; investigation; methodology; writing – original draft. **Chang Xu:** Conceptualization; data curation; investigation; methodology; writing – original draft. **Jianing Yang:** Data curation; investigation; methodology. **Yilan Song:** Investigation; methodology. **Li Li:** Data curation; investigation; methodology. **Jingzhi Jiang:** Data curation; formal analysis; investigation; methodology. **Liangchang Li:** Data curation; formal analysis; methodology; resources. **Chongyang Wang:** Funding acquisition; project administration; supervision; writing – review and editing. **Guanghai Yan:** Funding acquisition; project administration; supervision; writing – review and editing.

## FUNDING INFORMATION

This study was supported by the National Natural Science Foundation of China (82260007), the Jilin Province Science and Technology Department Project (20240404025ZP, 20240602100RC), the Jilin Provincial Department of Education Project (JJKH20240698KJ), and the Jilin Province Health Commission (2024A062).

## CONFLICT OF INTEREST STATEMENT

The authors declare that they have no competing interests.

## ETHICS STATEMENT

The study was reviewed and approved by the Ethics Committee of Yanbian University (protocol code: YB.No20210630b040, and date of approval: July 15, 2021). All experiments were performed following relevant named guidelines and regulations. The authors complied with the ARRIVE guidelines.

## Data Availability

The datasets adopted and analyzed in the study can be obtained from the corresponding author on reasonable request.
